# EANM practice guidelines for an appropriate use of PET and SPECT for patients with epilepsy

**DOI:** 10.1007/s00259-024-06656-3

**Published:** 2024-02-23

**Authors:** Tatjana Traub-Weidinger, Javier Arbizu, Henryk Barthel, Ronald Boellaard, Lise Borgwardt, Matthias Brendel, Diego Cecchin, Francine Chassoux, Francesco Fraioli, Valentina Garibotto, Eric Guedj, Alexander Hammers, Ian Law, Silvia Morbelli, Nelleke Tolboom, Donatienne Van Weehaeghe, Antoine Verger, Wim Van Paesschen, Tim J. von Oertzen, Pietro Zucchetta, Franck Semah

**Affiliations:** 1 Division of Nuclear Medicine, Department of Biomedical Imaging and Image-guided Therapy, Medical University of Vienna, Vienna, Austria; 2 Department of Nuclear Medicine, University of Navarra Clinic, Pamplona, Spain; 3 Department of Nuclear Medicine, Leipzig University Medical Centre, Leipzig, Germany; 4 Department of Radiology and Nuclear Medicine, Amsterdam UMC, Location VUmc, Amsterdam, The Netherlands; 5 Department of Clinical Physiology and Nuclear Medicine, University of Copenhagen, Blegdamsvej 9, DK-2100 RigshospitaletCopenhagen, Denmark; 6 Department of Nuclear Medicine, Ludwig Maximilian-University of Munich, Munich, Germany; 7 DZNE—German Center for Neurodegenerative Diseases, Munich, Germany; 8 Munich Cluster for Systems Neurology (SyNergy), Munich, Germany; 9 Nuclear Medicine Unit, Department of Medicine—DIMED, University-Hospital of Padova, Padova, Italy; 10 Université Paris-Saclay, CEA, CNRS, Inserm, BioMaps, 91401 Orsay, France; 11 Institute of Nuclear Medicine, University College London (UCL), London, UK; 12 Division of Nuclear Medicine and Molecular Imaging, Geneva University Hospitals, Geneva, Switzerland; 13 NIMTLab, Faculty of Medicine, University of Geneva, Geneva, Switzerland; 14 Center for Biomedical Imaging (CIBM), Geneva, Switzerland; 15 APHM, CNRS, Centrale Marseille, Institut Fresnel, Timone Hospital, CERIMED, Nuclear Medicine Department, Aix Marseille Univ, Marseille, France; 16 School of Biomedical Engineering and Imaging Sciences, Faculty of Life Sciences and Medicine, King’s College London & Guy’s and St Thomas’ PET Centre, King’s College London, London, UK; 17 Department of Clinical Physiology and Nuclear Medicine, Rigshospitalet, University of Copenhagen, Blegdamsvej 9, DK-2100 Copenhagen, Denmark; 18 Nuclear Medicine Unit, IRCCS Ospedale Policlinico San Martino, Department of Health Sciences (DISSAL), University of Genoa, Genoa, Italy; 19 Department of Radiology and Nuclear Medicine, University Medical Center Utrecht, Utrecht University, Utrecht, The Netherlands; 20 Department of Radiology and Nuclear Medicine, Ghent University Hospital, Ghent, Belgium; 21 Department of Nuclear Medicine and Nancyclotep Imaging Platform, CHRU Nancy, Université de Lorraine, IADI, INSERM U1254, Nancy, France; 22 Laboratory for Epilepsy Research, KU Leuven and Department of Neurology, University Hospitals, Leuven, Belgium; 23 Depts of Neurology 1&2, Kepler University Hospital, Johannes Kepler University, Linz, Austria; 24 Nuclear Medicine Department, University Hospital, Inserm, CHU Lille, U1172-LilNCog—Lille, F-59000 Lille, France

**Keywords:** Focal epilepsy, Temporal lobe epilepsy, Extratemporal epilepsy, Paediatric epileptic syndromes, Drug-resistant epilepsy, Interictal SPECT, Ictal SPECT, [^18^F]FDG PET, Neurotransmitter PET, Presurgical evaluation, Surgery, MRI, Imaging

## Abstract

Epilepsy is one of the most frequent neurological conditions with an estimated prevalence of more than 50 million people worldwide and an annual incidence of two million. Although pharmacotherapy with anti-seizure medication (ASM) is the treatment of choice, ~30% of patients with epilepsy do not respond to ASM and become drug resistant. Focal epilepsy is the most frequent form of epilepsy. In patients with drug-resistant focal epilepsy, epilepsy surgery is a treatment option depending on the localisation of the seizure focus for seizure relief or seizure freedom with consecutive improvement in quality of life. Beside examinations such as scalp video/electroencephalography (EEG) telemetry, structural, and functional magnetic resonance imaging (MRI), which are primary standard tools for the diagnostic work-up and therapy management of epilepsy patients, molecular neuroimaging using different radiopharmaceuticals with single-photon emission computed tomography (SPECT) and positron emission tomography (PET) influences and impacts on therapy decisions. To date, there are no literature-based praxis recommendations for the use of Nuclear Medicine (NM) imaging procedures in epilepsy. The aims of these guidelines are to assist in understanding the role and challenges of radiotracer imaging for epilepsy; to provide practical information for performing different molecular imaging procedures for epilepsy; and to provide an algorithm for selecting the most appropriate imaging procedures in specific clinical situations based on current literature. These guidelines are written and authorized by the European Association of Nuclear Medicine (EANM) to promote optimal epilepsy imaging, especially in the presurgical setting in children, adolescents, and adults with focal epilepsy. They will assist NM healthcare professionals and also specialists such as Neurologists, Neurophysiologists, Neurosurgeons, Psychiatrists, Psychologists, and others involved in epilepsy management in the detection and interpretation of epileptic seizure onset zone (SOZ) for further treatment decision. The information provided should be applied according to local laws and regulations as well as the availability of various radiopharmaceuticals and imaging modalities.

## Preamble

The EANM is a professional non-profit medical association that facilitates communication worldwide among individuals pursuing clinical and research excellence in NM. The EANM was founded in 1985. These guidelines are intended to assist practitioners in providing appropriate NM care for patients with focal epilepsy. They are flexible rules or requirements of practice and are not intended, nor should they be used, to establish a legal standard of care. The ultimate judgement regarding the propriety of any specific procedure or course of action must be made by medical professionals taking into account the unique circumstances of each case. Thus, there is no implication that an approach differing from the guidelines, standing alone, is below the standard of care. To the contrary, a conscientious practitioner may responsibly adopt a course of action different from that set out in these recommendations when, in the reasonable judgement of the practitioner, such course of action is indicated by the condition of the patient, limitations of available resources, or advances in knowledge or technology subsequent to this publication. The practice of medicine involves not only the science but also the art of dealing with the prevention, diagnosis, alleviation, and treatment of disease. The variety and complexity of human conditions make it impossible to always reach the most appropriate diagnosis or to predict with certainty a particular response to treatment. Therefore, it should be recognized that adherence to these guidelines alone will not ensure an accurate diagnosis or a successful outcome. All that should be expected is that the practitioner will follow a reasonable course of action based on current knowledge, available resources, and the needs of the patient to deliver effective and safe medical care. The sole purpose of these guidelines is to ensure that the practitioner delivers effective and safe medical care by following a reasonable course of action based on current knowledge, available resources, and patient needs.

## Purpose

These EANM practice guidelines, written for NM healthcare professionals and other specialists who are involved in epilepsy management, should support the optimal use of NM procedures in epilepsy imaging in children and adults based on current knowledge. The purposes of these recommendations are as follows:

- To assist in understanding the role and challenges of radiotracer imaging for epilepsy.
- To provide practical information for performing NM imaging procedures for patients with focal epilepsy.
- To provide an algorithm for selecting the most appropriate NM imaging procedure in specific clinical situations.
- To provide information regarding radiotracers that can lead to a better understanding and characterization of epilepsy.

## Introduction

Epilepsy is a chronic non-communicable disease of the brain that affects people of all ages and one of the most frequent neurological conditions with an estimated prevalence of more than 50 million people worldwide and an annual incidence of two million [1]. Epilepsies/epileptic syndromes are classified as focal, generalized, combined focal and generalized, or unknown, with focal epilepsy being the most frequent form [2]. A range of aetiologic groups is defined with emphasis on those that have implications for treatment. In patients with focal epilepsy, MRI is the first imaging investigation, which enables the clinician to decide if there is a structural aetiology for the patient’s epilepsy, such as hippocampal sclerosis, tumour, focal cortical dysplasia (FCD), haemorrhage, or other structural lesions. The five additional aetiologic groups are defined as genetic, infectious, metabolic, immune, or unknown. A patient’s epilepsy also may be classified into more than one etiologic category. Moreover, the etiologies are not hierarchical, and the importance given to the patient’s etiological group may depend on the circumstance, as given for instance in patient who has both a structural and a genetic or metabolic aetiology with different potential treatment options [2].

Pharmacotherapy with ASM is the initial treatment of choice for the vast majority of patients with epilepsy. Approximately 30% , however, do not respond to two ASMs and are considered drug resistant [3–5]. This is mainly true for focal epilepsies [6]. Similar findings are observed in children and adolescents with approximately 20% of drug resistance with risk for poor long-term cognitive and psychosocial outcomes together with poor quality of life [7, 8]. In patients with drug-resistant focal epilepsy, epilepsy surgery is promising depending on the epilepsy type with approximately 65% of patients becoming seizure free [9–11]. If the source of epilepsy is correctly detected and outside of eloquent areas, surgery of epileptogenic zone is overall safe, successful, and cost-effective [12].

Beside scalp video/EEG telemetry, structural MRI, neuropsychological and neuropsychiatric assessment, Wada test, functional MRI, NM imaging procedures, and intracranial EEG electrodes are of additional value depending on the epilepsy type. The development of molecular neuroimaging with interictal [^18^F]fluorodeoxyglucose ([^18^F]FDG) positron emission tomography (PET), ictal perfusion SPECT, or ictal subtraction perfusion SPECT (interictal SPECT fused, normalized, and subtracted from ictal SPECT) has influenced and impacted the presurgical management of epilepsy patients, not only for the investigation of non-lesional, but also lesional epilepsy regarding postsurgical seizure outcome over the years. In both adults and children without any visible brain lesion on MRI or in others with multifocal structural cerebral abnormalities such as hemispheric or multi-lobar cortical dysplasia, polymicrogyria, or localized stroke, these functional imaging tools are especially useful to localize the seizure onset for a tailored resection preserving motor, visual, language, or temporal lobe functions [13]. To date, there are no literature-based praxis recommendations for the use of NM imaging procedures in epilepsy. It is the aim of this “guideline” to fill this gap.

## Precautions

### Imaging pregnant or potentially pregnant adolescents and women with ionizing radiation

The use of radiopharmaceuticals is generally contraindicated in pregnancy. However, if clinically necessary, a decision to perform imaging in cases of known or suspected pregnancy should be based on an analysis of the benefits versus the possible risks to the foetus, as decided by a multidisciplinary team [14, 15]. Otherwise, it should be postponed after pregnancy. Proper institutional and local guidelines should be followed.

### Breastfeeding women

In breastfeeding women, it is recommended to consult the International Commission on Radiological Protection (ICRP) Publication 128: Radiation Dose to Patients from Radiopharmaceuticals: A Compendium of Current Information Related to Frequently Used Substances [16]. Although interruption of breastfeeding is not essential in the absence of free pertechnetate in ^99m^Tc-labelled radiopharmaceuticals, a pause of 24 h is recommended in some summaries of product characteristics of ^99m^Tc-labelled radiopharmaceuticals with marketing authorization [17]. There is no information ^11^C-labelled pharmaceuticals described in these guidelines, including L-([^11^C]methyl)methionine ([^11^C]MET), regarding excretion of radioactivity into breast milk. Nevertheless, due to their short physical half-life an interruption is not essential. For [^18^F]FDG, interruption of breastfeeding is not recommended; for other ^18^F-labelled compounds described here recommendations concerning the duration of a pause in lactation are not available, but discontinuing breastfeeding with milk discard for 12 h after tracer injection should be considered [16, 18].

### Intradepartmental care

A continuous supervision of epilepsy patients, especially prior and during the procedure, is essential to guarantee inter-ictal imaging, a seizure-free examination, and to ensure the safety of these at-risk patients. The healthcare professionals have to be instructed regarding potential ictal signs occurring during the uptake phase of some radiopharmaceuticals and imaging phase. ASM and other medications needed should be available in the division or scanning room. The occurrence of a seizure or status epilepticus needs to be documented and taken into consideration when interpreting the functional images, if relevant.

### Imaging of children and adolescents in company of parents or caregivers

Following institutional and local guidelines and to reduce the anxiety of the child and the parents or caregivers, they have to be informed regarding the radiopharmaceutical used and its specific indication as well as procedural details of scanning. Moreover, they have to be instructed to notify the medical staff when an ictal sign occurs prior or during the uptake phase of different tracers.

### Imaging of epilepsy patients under anaesthesia

In the case of inability or non-cooperation regarding the requested examination anaesthesia may be necessary in adults as well as paediatric epilepsy patients, even if it has influence on brain metabolism and perfusion [19] and especially if anaesthesia is administered less than 30 min from the injection of the tracer [20]. Beside following the institutional and local guidelines the anaesthesia team has to be informed regarding tracer behaviour, radiation protection, and scanning procedure.

## Qualifications and responsibilities of personnel

NM physicians, technologists, nurses, and all other healthcare professionals involved in performing and reporting epilepsy imaging should be qualified according to applicable laws and regulations, and individual responsibilities should be clearly documented.

## Useful clinical information for optimal imaging and interpretation

A request for epilepsy imaging must be submitted to a NM physician. It should provide data regarding the clinical history of the patient, with the type of seizure and frequency, dominant hemisphere, current and previous medications, EEG or/and video-telemetry data, and previous MRI imaging, to allow the NM physician responsible to assess the indication for the scan adequately, as well as to interpret the images. The patient is also questioned (if possible) for relevant information that may help interpret the findings (e.g., time of last seizure, current medication). ASM and other required medications are permitted although cautions should be used with certain drugs such as benzodiazepines.

Timing of PET and SPECT imaging, especially for interictal perfusion SPECT and [^18^F]FDG PET, has to be documented according to the time of last seizure. If clinically possible, high- quality interictal SPECT and [^18^F]FDG PET imaging in epilepsy patients should be performed at least 24 h after focal aware seizures and 48 h after focal impaired awareness and focal-to-bilateral tonic-clonic seizures [21].

## Perfusion SPECT imaging

The major originality of SPECT imaging remains its ictal imaging value by measuring the regional cerebral blood flow associated with epileptic seizures [22]. Actually, the radiopharmaceutical administration can be performed during an epileptic discharge, with a brain uptake irreversibly completed in 1 to 2 min [23]. During an ictal scan, the brain regions involved in seizure generation and early propagation demonstrate increased perfusion, with all hyperperfused regions representing an electrically connected epileptic network [23]. By contrast, most epileptic networks are hypoperfused during inter-ictal state. Ictal SPECT presents a sensitivity of 73% and specificity of 75% while inter-ictal SPECT has a much lower localisation value with 50% of sensitivity and 75% of specificity [24]. Moreover, SPECT has higher performances in detection of epileptic networks in temporal lobe epilepsy (TLE) in comparison to extra-temporal lobe epilepsy (ETLE) [25]. Notably, ictal SPECT is useful for lesional epilepsy, suitable in children with focal refractory epilepsies while being a strong predictor of surgical success [26]. With an odds ratio of 0.37, ictal SPECT is also a favourable surgical outcome predictor in non-lesional epilepsy [27]. Subtraction of ictal and inter-ictal SPECT co-registered to MRI (SISCOM) has been shown to improve the sensitivity and specificity of seizure related perfusion networks. SISCOM not only shows regions of hyperperfusion, but also hypoperfusion. These ictal-network related perfusion changes have been well described during focal impaired awareness seizure in mesial temporal lobe epilepsy with hippocampal sclerosis with ictal hyperperfusion of the temporal lobe ipsilateral to the seizure focus, the border of the ipsilateral middle frontal and precentral gyrus, both occipital lobes and small regions the contralateral postcentral gyrus. The frontal lobes, contralateral, the posterior cerebellum and the ipsilateral precuneus showed hypoperfusion [28]. The diagnostic performances of SISCOM are potentially further enhanced when using statistical SPECT processing methods. In this line, studies found that SISCOM localization sensitivity was higher than 90% in temporal lobe seizures [29], but lower in ETLE, with a reported sensitivity of 70% [30]. SISCOM provides useful information for seizure localization in patients with focal cortical dysplasia, even with normal MRI [31]. From a practical clinical value in preoperative evaluation, SISCOM was compared with either MRI, [^18^F]FDG PET, ictal EEG, or EEG-fMRI or combined modalities. For instance, hyperperfusion patterns in SISCOM images were localized more often than with side-by-side SPECT evaluation (71.0 vs. 47.4%), whereas SISCOM images led to at least a concordant or only slightly lower focus detection rate than [^18^F]FDG PET, MRI, and EEG modalities alone or combined [32–37]. Interestingly, if SISCOM localization is concordant with the surgical resection site on other traditional focus localization techniques, then postoperative outcomes are expected to be favourable [30, 38–42], with a seizure-free odds ratio around 3-times higher in concordant than in non-concordant SISCOM patients [43].

However, spatial resolution of SPECT is limited. Consequently, inter-ictal studies in focal epilepsies are often performed with PET imaging which leads to a better sensitivity in detection of epileptic networks. Albeit studying metabolism and perfusion are different physiological processes [44], they are in most circumstanced coupled. It should nevertheless be reported the recent instrumentation development, particularly Cadmium-Zinc-Telluride (CZT) cameras. These CZT cameras provide more than twofold increase in count sensitivity, as compared with the Anger camera, as well as better image resolution for brain imaging [45].

### Technique: ictal, interictal, ictal subtraction SPECT

The reader should refer to the “EANM procedure guidelines for brain perfusion SPECT using ^99m^Tc-labelled radiopharmaceuticals, version 2” for a detailed report of the recommended principles for image interpretation and support from semi-quantification strategies [46]. Here we will provide only additional details referring specifically to SPECT brain imaging in epilepsy.

A. Procedures
  It should be necessary to differentiate SPECT imaging performed during ictal and interictal phases. During an “ictal” scan, the brain regions involved in seizure generation and early propagation demonstrate increased perfusion, while most epileptic networks are hypoperfused during inter-ictal state.
  ^99m^Tc-radiolabelled tracers such as HexaMethylPropyleneAmine Oxime ([99mTc]Tc-HMPAO) or Ethyl Cystine Dimer ([99mTc]Tc-ECD) are currently used according to the product manufacturing recommendations with an injected activity, in adults, of typically 740 MBq [24].
  For ictal SPECT studies, the tracer should be injected as soon as possible after seizure onset, ideally within 20 s (via an intravenous line placed previously). Patients should have continuous video-EEG monitoring [46]. The best results are obtained when injection occurs during focal impaired awareness seizures. It is recommended that prepared syringes be stored in the epilepsy monitoring unit to ensure the quickest possible injection. The use of an automatic injector in ictal SPECT demonstrated significant clinical value by increasing the number of single localizing foci in comparison to scans with a manual injection by decreasing latency time [47, 48].
  SPECT image acquisition can start 30 to 90 min after tracer injection with acquisition duration of about 20 min. For inter-ictal perfusion SPECT studies, EEG monitoring is recommended, if available, during tracer injection to exclude potential ictal activity especially in the first 2 min of the uptake phase of the radiopharmaceutical. Inter-ictal studies may add useful information to ictal studies. However, these cannot be recommended as a sole diagnostic procedure for focus detection [46].
B. Interpretation
  Interpretation of ictal and inter-ictal perfusion SPECT is based on a visual analysis, which can be improved by the addition of semi-quantitative analyses and co-registration to MRI.
  As already noted, SISCOM is particularly useful; this has been shown to improve the sensitivity and specificity of seizure localization networks only demonstrating hypoperfusion during inter-ictal scan [49]. Knowledge of the injection timing and injected seizure type is important for a correct interpretation of SISCOM images [50]. The most commonly used thresholds for SISCOM are z=+1.5 and z=+2 [51].

### Radiation doses

As the ICRP publication 128 reported in the adult population, the absorbed dose per unit of activity (without considering CT attenuation) administered (mGy/MBq) for urinary bladder wall is 5E-02 for [^99m^Tc]Tc-ECD and 2.3E-02 for [99mTc]Tc-HMPAO resulting in an effective dose of 7.7E-03 mSv/MBq for [^99m^Tc]Tc-ECD and 9.3E-03 mSv/MBq for [^99m^Tc]Tc-HMPAO. In children (above 5 years of age) the effective dose raises to 1.5E-02 mSv/MBq for [^99m^Tc]Tc-ECD and 1.7E-02 mSv/MBq for [^99m^Tc]Tc-HMPAO [16].

## [^18^F]FDG brain PET imaging

As a radiolabelled glucose analogue, in resting condition and interictal phase [^18^F]FDG PET typically shows reduced radiotracer uptake (hypometabolism) in the SOZ and the propagation zones beside a global decrease of cerebral grey matter glucose metabolism of around 10–25% compared with control subjects [52, 53]. This pattern has been addressed to various pathophysiological mechanisms, such as neuronal loss, reduction in synaptic density, and other epileptic-related dysfunctions, including post-ictal vascular changes or post-ictal depression. Factors like epilepsy duration and seizure frequency, ASMs, delay between the last seizures, type of the last seizure (e.g., generalized/unusual seizure) may be also responsible for the reduced uptake of [^18^F]FDG [54–60].

Interictal metabolic imaging with [^18^F]FDG helps the non-invasive detection of epileptogenic focus with higher sensitivity than inter-ictal perfusion SPECT, reflecting seizure-related changes in cerebral functions [44]. In fact, [^18^F]FDG PET visually demonstrates not only the SOZ, but the whole irritative zone (e.g., the SOZ and subsequent neural networks involved in the generation of inter-ictal paroxysms) [61, 62]. Moreover, [^18^F]FDG hypometabolism beyond the presumed SOZ is reported with a similar frequency in both TLE and ETLE surgical candidates with significant predictive value for surgical outcome [63].

A step forward in the diagnostic clinical use of [^18^F]FDG brain PET has been given both by the introduction of digital PET, which provides better image quality, diagnostic confidence and accuracy than analogue PET [64], and from hybrid PET/MRI systems, thanks to their simultaneous multimodal acquisition resulting in a perfect image fit with good surgical outcome [65, 66]. Moreover concordance of PET/MRI and magnetoencephalography (MEG) showed improvement of the presurgical localization of the epileptogenic focus [67]. In non-PET/MRI systems, the co-registration between PET/CT and MRI can be performed using dedicated software packages [68].

Over the years, [^18^F]FDG PET has become part of the systematic pre-surgical assessment of drug-resistant epilepsies. In decision making studies it showed to be useful for surgery selection in up to 47% and most beneficial in TLE (58–69%) compared to ETLE (23–44%) [69, 70]. It can also contribute to guide in the search for subtle cortical dysplasia or the placement of intracranial electrode [71], as well as to the assessment of patients with multifocal abnormalities to determine which is most likely to cause the seizure as the epileptogenic focus [72]. Moreover, [^18^F]FDG PET helps to re-interpret the negative MRI, detecting lesions missed at the first look, for example cases of focal cortical dysplasia in adults or immature myelination and poor grey to white matter differentiation in infants [73, 74]. It was also shown to be a valuable non-invasive method to guide intracranial electrode placement, and can also reduce the number of patients requiring invasive EEG [75]. Finally, [^18^F]FDG PET can be useful for the evaluation of the functional status of the rest of the cortex, which could serve as a predictor of the cognitive outcome after resection [76].

### Lesional and non-lesional temporal lobe epilepsy in adults

The role of [^18^F]FDG PET in the assessment of TLE is of paramount importance in neuro-imaging. This has a reported pooled sensitivity and specificity of 79–95% in detection of the epileptic brain region in cases of TLE [63, 77]. Different groups of electro-clinical patterns, accordingly to distinct patterns of TLE, showed good accordance with inter-ictal [^18^F]FDG PET hypometabolisms at group level [61]. Further concordance with stereoelectroencephalography (SEEG) findings and inter-ictal [^18^F]FDG PET hypometabolism were also reported at the group and individual level [78]. It is however worth to underline again that temporal lobe hypometabolic regions often extend beyond the presumed epileptogenic zone, likely reflecting the degree of cerebral dysfunction that may be due to loss of synaptic inputs related to seizures. A gradient of [^18^F]FDG hypometabolism is here described between non-involved zone and propagation, epileptogenic, and lesional zones with nevertheless good performance in defining the epileptic zone [62]. These findings are also consistent with the current notion of epileptogenic and propagation networks [79] and allow to consider [^18^F]FDG metabolism as a network rather than a combination of regional metabolic measurements impacting on surgical outcome [80] with also surgical failure characterized by relatively high extratemporal hypometabolism on both sides [81–83].

In general, it is not infrequent to see other regions of diffuse glucose hypometabolism in epileptic patients, the significance of which is not always simple to determine. As known these patients, due to seizure and generally abnormal electric (consequent) metabolic activations of certain areas, may have interictally different neurological conditions. In patients with unilateral TLE, prefrontal asymmetric interictal hypometabolism is described to be associated with mild cognitive impairment [84], similarly, the presence of bitemporal glucose hypometabolism reflecting memory deficit with higher risk of postoperative memory decline [85]. Moreover, the left temporal pole seems also to be involved in lexical and semantic retrieval of knowledge of famous persons [86], the left temporo-occipital areas with potential deficit on word findings [87]. Hypometabolism associated with the ipsilateral insular cortex may correlate with emotional or somesthetic symptoms [88]. Evidence of contralateral thalamic hypometabolism is described to be associated with poorer surgical outcome compared to ipsilateral thalamic hypometabolism usual seen in mesial temporal lobe epilepsy [89]. More recently, it has been shown that cognitive impairment correlates with extratemporal hypometabolism, involving the mesial frontoparietal networks implicated in the default mode network and suggesting a disconnection with the affected hippocampus [90].

### Lesional and non-lesional extra-temporal lobe epilepsy in adults

In ETLE, [^18^F]FDG PET performs with a lesser (as compared to TLE) sensitivity of approximately 55% in localizing the epileptogenic zone in retrospective studies [91]. One prospective trial, however, reported a sensitivity and specificity of 80% and 95% in a presurgical setting [63]. When focal hypometabolism is present, this means a significant positive indicator of postoperative seizure freedom when used as a means of secondary diagnostic imaging. Especially the combination of visual together with quantification tools provides additional prognostic outcome [27, 63, 92]. Sometimes [^18^F]FDG pattern in patients with ETLE can be misinterpreted because of also prominent hypometabolism in the temporal lobe [91]. Other factors which may be associated with a poorer post-surgical prognosis is hypometabolism remote from the epileptogenic zone [63, 93]. In frontal lobe epilepsy the sensitivity of [^18^F]FDG PET is reported to be higher (73%) in patients with structural lesions compared to patients without structural lesions on MRI (36%) [94]. In parietal epilepsy no differences were seen in PET focus localization frequency in seizure free compared to non-seizure free patients after surgery, although MRI abnormality and concordance of different diagnostic modalities such as PET, ictal SPECT, and ictal EEG were associated with high seizure-free rate [95]. Moreover, functional neuroimaging may also aid to confirm and complementary an occipital epileptogenic focus, with normal MRI with better performance of PET compared to SPECT to define the epileptogenic zones [96, 97]. Finally, co-registering PET images with MRI or using PET/MRI serves as additional tool to enhance the results for the detection of lesions on MRI in ETLE patients providing additional information on good prognostic surgical outcome or increased confidence in the original clinical readings [74, 98–101].

Although [^18^F]FDG hypometabolism is the most seen imaging pattern in interictal PET, hypermetabolic cortical or subcortical foci can sometimes be observed. Grey matter heterotopic tissue, a neuronal migration disorder as malformation of cortical development (MCD) may be present as hypermetabolism surrounded by white matter with only low [^18^F]FDG uptake [102, 103]. Others discuss hypermetabolic brain abnormalities as pathological neuronal hyperactivity predisposed to epileptic discharge generation or as a network of activated inhibitory circuits preventing the spread of epileptic discharge originating from a localized epileptogenic zone [101, 104–106].

### Paediatric focal epilepsy and syndromes

In children and adolescents, the aetiology of focal epilepsy is multifactorial and includes other entities such as tumours (especially low grade epilepsy associated brain tumours—LEAT), birth-related lesions (stroke or haemorrhages), or MCD rather than mesial temporal sclerosis [107]. A number of MCDs are caused by an underlying genetic defect, seen as an isolated disorder, or associated with a wide variety of neurological symptoms such as developmental delay and/or motor abnormalities and extra-neurological features [108, 109]. Based on their MCD phenotype they could variably present as a focal irregularity of cortical morphology and thickness as observed in FCD or as an excessive number of abnormally small cerebral gyri with cortical overfolding as seen in polymicrogyria (PMG) or as hemimegalencephaly (a congenital unilateral disorder with cortical malformation). [^18^F]FDG PET may provide important information together with structural and other functional imaging techniques beside EEG regarding the localization of epileptogenic foci and its extension for treatment planning and prediction of postsurgical seizure outcome, avoiding incomplete resection [110]. The lesions will typically exhibit hypometabolism with some exceptions as discussed elsewhere in the text. The additional value of [^18^F]FDG PET in paediatric epilepsy cases is the possibility of defining the lesion with higher sensitivity (and usually larger extent) as compared to MRI, or localizing lesions in patients with negative or doubtful findings on MRI, especially in patients with FCD type 2 [99, 111–113]. Moreover, in situations of multilobar drug-resistant foci of epilepsy, a well-defined surgical approach such as the posterior disconnection or functional hemispherotomy (HE) is possible [114–116]. To achieve a good post-surgical epileptogenic outcome in these patients with potential of improvement of mental and physical development, the epileptogenic lesion should not extend beyond the confines of the disconnected cerebral volume. Subtle MRI abnormalities more widespread than the clear-cut lesion or MRI abnormalities in the contralateral remaining hemisphere may influence the surgical outcome [116, 117]. [^18^F]FDG, in the setting of bilateral focal metabolic abnormalities, has been therefore recognized as an important additional imaging tool [118, 119] and moreover described as an independent predictor of seizure recurrence after HE [117].

For epilepsy syndromes (ES) [^18^F]FDG PET also showed to be of interest especially in the setting of pre-surgical evaluation and postsurgical outcome estimation. ES are described as “characteristic clusters of clinical and electroencephalographic features, often supported by specific etiological findings (structural, genetic, metabolic, immune, and infectious)” and have been recently defined by the International League Against Epilepsy (ILAE) Task Force on Nosology and Definitions by dividing syndromes into typical age at onset, and based on seizure and epilepsy type in association with developmental and or epileptic encephalopathy or progressive neurological deterioration [120].

*Tuberous Sclerosis* (TSC), an autosomal-dominant multiorgan disorder caused by TSC1 gene (hamartin; chromosome 9q34) or TSC2 gene (tuberin; chromosome 16p13.3) mutations, is characterized by the development of hamartomatous lesions in various organs, including the brain. Epilepsy is drug resistant in 50–80% of these patients with poor cognitive outcome [121]. Early epilepsy surgery shows some benefit with resecting the main epileptogenic tuber [122]. Beside perfusion SPECT imaging [123, 124] [^18^F]FDG PET can also play a valuable role by non-invasively lateralizing and localizing the main epileptogenic tuber as area of glucose hypometabolism [125, 126], often more extended than observed with MRI indicating dysplastic cortex [127].

*Sturge-Weber Syndrome* is a rare sporadic neurocutaneous syndrome with seizure development present in up to 90% of these children. In these cases [^18^F]FDG usually shows larger area of abnormal cortex as compared to MRI. Although the involved cortex is hypometabolic interictally, the presence of cortical hypermetabolism in young children may be an imaging marker of subsequent severe epilepsy, requiring early surgical intervention [128–130].

*Lennox-Gastaut Syndrome* is characterized by a profoundly impairing developmental and epileptic encephalopathy with poor responsiveness to antiepileptic medication. Few publications are available regarding the utility of [^18^F]FDG PET in this disorder, with description of different tracer uptake patterns [131] and possible value in surgical treatment planning in patients with unilateral focal hypometabolism based on good concordance of PET and ictal EEG findings [132].

*Hemimegalencephaly* is a severe congenital malformation with unilateral enlarged and defectively developed hemisphere in the children affected by intractable seizures. Early hemisphere disconnection may lead to seizure control and adequate cognitive development. Therefore, the evaluation of the remaining contralateral hemisphere is important. [^18^F]FDG PET showed to be useful for assessing the functional integrity of the contralateral hemisphere and supporting in prediction of cognitive outcome [133].

*Rasmussen Encephalitis* is a form of chronic focal encephalitis associated with inflammation and progressive atrophy of a single hemisphere. During the early stages of the disease, [^18^F]FDG but also perfusion SPECT imaging can detect functional abnormalities as both hyper- and hypometabolism depending on the seizure status during the examination and may be helpful in identification of inconclusive MRI findings of the affected side [134–136].The most useful aspect of [^18^F]FDG PET in this setting is probably the exclusion of a bi-hemispheric involvement [137].

In general, brain [^18^F]FDG PET images of paediatric epilepsy patients are—as those of adult patients—mostly assessed visually. As the normal pattern of cerebral glucose consumption shows a strong age dependency in children/adolescents [112–115], such an approach, however, may be particularly subjective and user dependent. Therefore, a comparison of the respective experiences across different centres is difficult and may be a reason for the variable performance of [^18^F]FDG PET reported in the paediatric epilepsy literature. Software-based quantifying systems may be useful for better definition of the epileptic zone in paediatric patients with focal epilepsy. Such systems have so far been tested, however, only in children older than 6–8 years. Semi-quantitative data should, as such, be interpreted carefully, considered abnormal if significantly outside the range of normal data obtained from age-matched controls and only in combination with the visual inspection, as also recommended by others [138].

### Principles and techniques for image interpretation

The reader should refer to the recently published “EANM Procedure Guidelines for Brain PET Imaging using [^18^F]FDG, version 3” for a detailed report of the recommended principles for image interpretation and support from semi-quantification strategies [20]. Here we will provide only additional details referring specifically to [^18^F]FDG brain imaging in epilepsy.

A. Visual interpretation
  The images should be critically examined prior to interpretation for presence of movement artefacts, the risk of which is higher in the epileptic population as seizures might occur during the acquisition.
  Similarly, alignment of the images used for attenuation correction (CT for PET/CT, or µ-maps or specific sequences for PET/MRI) with the emission images should be systematically checked as mismatches can lead to erroneous interpretation. Furthermore, especially in very young children (therefore with possible incomplete myelinization) MRI derived µ-maps should be checked for possible biases related to erroneous segmentation.
  Attenuation artefacts might occur in the presence of electrodes for EEG monitoring and might have an impact on quantification that should be taken into account interpreting the images [139].
  The visual analysis should systematically evaluate all brain regions, using asymmetry as the main criterion for abnormality. This visual analysis can be refined using an anatomy-corrected asymmetry index image [140]. One should be careful not to miss bilateral and symmetric hypometabolism [53].
  The visual analysis should also systematically evaluate the whole brain cortex after fusion of [^18^F]FDG images with a recent MRI (or CT in patients with contraindications for MRI) scan, preferably obtained near contemporary especially in children under 6 years old. The added value of PET and MRI image coregistration has been consistently confirmed across different patient series [74, 99, 141] and could potentially reduce confounding factors (e.g., fissures, CSF spaces, or cortical damages).
  PET/MRI systems provide images acquired in the same reference space and thus automatically fused without the need of specific software for image registration (however, alignment of attenuation and emission data still needs to be checked especially if both have not been acquired at the same time). PET/MRI systems have been specifically employed in epilepsy, showing the ability of this tool to provide relevant diagnostic information across different modalities, including also electroencephalography [65, 101, 142–146].
B. Semi-quantification and automated analysis
  The added value of automated tools for the interpretation of images has been reported in the literature [68, 94, 147, 148]. The majority of tools have been validated and used for the evaluation of neurodegenerative patterns, and reference populations included or publicly available are usually composed of older individuals [20]. One of the main challenges is thus the use of a reference database and a pipeline adapted to the age of the subject investigated, mainly for children and adolescent patients: the limitation associated with a limited matching should thus be taken into account [149–151]. In addition to age, PET data acquisition and reconstruction differences should be considered when making comparisons against a normal database.
  The results of software for semi-quantification and voxel-wise analysis should be used to guide a “second-look” visual analysis to detect subtle asymmetries or focal reductions.
  PET data post-processing taking into account partial volume effects might be useful in this indication, but has not been extensively validated so far [152, 153].
  A number of automated methods, including deep-learning and artificial intelligence approaches, have been more recently developed and reported for detection of abnormalities in epilepsy and represent promising developments; however, none has been clinically validated yet [153–156].
C. Final report
  For the final report see also the EANM Procedure Guidelines for Brain PET Imaging using [^18^F]FDG, version 3. [20] Moreover, it is important to combine the visual and if available semi-quantitative analysis, pointing out the presence of abnormalities and, in case of hypo- and/or hypermetabolism, their location and extent, as this information has been shown to carry not only diagnostic but also prognostic value for the surgical treatment of both TLE and ETLE [63].

### Radiation doses

Based on activities administered between 125 and 250 MBq and based on the EANM dosage card v.01.02.20 in children as recommended by the EANM Procedure Guidelines for Brain PET Imaging using [^18^F]FDG, version 3, an effective dose of 0.019 mSv/MBq in adults and 0.056 mSv/MBq in children can be assumed [20, 157]. With the possibility of lowering activities, probably by a factor of 2 or more, with the use of high sensitivity digital systems (TOF < 400ps) and/or long axial field of view systems, the effective dose will be reduced via reduced tracer dose administration, but limited data are available at present to give clear recommendations [20].

## Other tracers for epilepsy imaging

In this section, additional radioligands are presented, which are complementary, clinically relevant in the pre-surgical work-up of epilepsy or may contribute to a more detailed characterization and understanding of this disease.

### [^11^C]/[^18^F]flumazenil

Gamma-aminobutyric acid (GABA) is the most important inhibitory neurotransmitter in the central nervous system. Flumazenil blocks the benzodiazepine sites on GABA-A receptors and thus antagonizes the action benzodiazepines have on the central nervous system. Flumazenil was introduced in 1987, and over time it was used as an antidote for the treatment of benzodiazepine overdoses. In epileptogenic regions a reduced level of benzodiazepine receptors was found by some authors [158], others reported false lateralization [159]. The PET radioligand [^11^C]flumazenil ([^11^C]FMZ) is selective for the GABA-A receptor subunits α1–3 and α5 [160]. [^11^C]FMZ binding is reduced in the hippocampi and other temporal lobe regions of individuals with refractory TLE even in the presence of a normal MRI [161] and in cryptogenic frontal epilepsy [159]. Similarly [^11^C]FMZ-PET has been used for detection of neocortical extratemporal epilepsy [162]. PET acquisition is usually performed 20 to 40 min after the injection of 370–555 MBq of [^11^C]FMZ. However, full quantification with an arterial input function leads to higher detection rates of the known epileptogenic lesion hippocampal sclerosis compared to summed static images [163]. Treatment with benzodiazepines, γ-vinyl-GABA and tiagabine should be disrupted one month before PET acquisition. In a recent meta-analysis, [^11^C]FMZ displayed an overall sensitivity of 62% (95% CI: 49–73%) and specificity of 73% (95% CI: 59–84%) for the localization of the epileptogenic zone and to provide evidence for practitioners’ clinical decision-making [77]. The area of decreased [^11^C]FMZ binding is often smaller than that of [^18^F]FDG hypometabolism (48%) or larger than that of the MRI abnormality (28%) [159]. Moreover, [^11^C]FMZ binding is positively correlated with interictal interval, and negatively correlated with seizure frequency [164, 165]. Nevertheless, the added value of FMZ as compared to [^18^F]FDG-PET has not been fully demonstrated with also false lateralization of epileptic focus reported by FMZ-PET in a study using quantification with the partial saturation method [166]. Moreover, the use of [^11^C]FMZ in clinical practice has been limited by its short half-life and necessitating an on-site cyclotron for production. ^18^F-labelled FMZ tracers might be the alternative of choice for patients with refractory epilepsy, but are not routinely in clinical use [167]. There are some compounds available, including 5-(2′-[^18^F]fluoroethyl) flumazenil ([^18^F]FEFMZ), 3-(2′-[^18^F]fluoroflumazenil ([^18^F]FFMZ), 5-(2′-[^18^F]fluoroethyl)flumazenil ([^18^F]FEF), and [^18^F]flumazenil ([^18^F]FMZ). Compared to [^11^C]FMZ, [^18^F]FEFMZ has lower receptor affinity and higher nonspecific binding due to faster metabolism and faster kinetics. [^18^F]FFMZ also has faster kinetics than [^11^C]FMZ [167]. Static images can be obtained as early as 10 to 15 min of the injection 197 to 370 MBq of [^18^F]FMZ. Binding of FMZ is, however, more indicative of the expression of α1 rather than α5 subunits of the GABA-A receptor with potential impact of benzodiazepine receptor binding medications [168]. New tracers like [^11^C]Ro15-4513 have approximately nanomolar affinity for GABAA receptors, with approximately 10–15 times higher affinity for those receptors containing α5 than for those that do not. Clinical validity has not been demonstrated for [^11^C]Ro15-4513. However, a recent study in MRI-negative TLE produced, from a single injection, two parametric maps (one each for α1 and α5) via bandpass analysis showing bilaterally increased α5 binding, likely linked to poor memory function, and ipsilaterally decreased α1 binding as with flumazenil radiopharmaceuticals [169].

### [^11^C]methyl-L-tryptophan

Alpha-[^11^C]methyl-L-tryptophan ([^11^C]AMT) PET shows increased tracer uptake interictally in epileptogenic tubers only of patients with tuberous sclerosis (TSC1 and TSC2) and in dysplastic cortex with an excellent agreement in seizure focus lateralization between ictal scalp EEG and [^11^C]AMT. It is also found to be more localizing in patients with non-lateralized ictal EEG for a successful surgical intervention. For PET imaging, fasting for 6 h to obtain stable plasma tryptophan and large neutral amino acid levels is advised with image acquisition 25 min after tracer injection (3,7 MBg/kg) under sedation, if needed. To achieve a seizure-free outcome after surgery with an accuracy of ~83% for the detection of epileptogenic tubers a cut-off threshold uptake ratio relative to normal-appearing cortex of 1.02 is recommended, evaluated based on image fusion with MRI for quantitative [^11^C]AMT PET assessment [170–172]. The tracer uptake appears to be due to the activation of the kynurenine pathway, leading to the production of quinolinic acid, a proconvulsant [173]. With a short physical half-life of ^11^C, the radiation exposure is low and comparable to other ^11^C-labelled amino-acid tracers. Because facilities with an on-site cyclotron are needed, [^11^C]AMT is only used in few NM units.

### Radiolabelled amino-acid compounds

Published data within radiolabelled amino acid (AA) compounds have primary been using [^11^C]MET, O-(2-[^18^F]fluoroethyl)-L-tyrosine ([^18^F]FET) and also 3,4-dihydroxy-6-[^18^F]fluoro-L-phenylalanine ([^18^F]FDOPA). The performance, interpretation, and reporting of PET scans follow the principles for glioma as outlined in recently published procedure guidelines by the clinical and nuclear imaging societies [174, 175]. The increased AA uptake in glioma is caused by an upregulation of the L-aminoacid transporter (LAT). The primary clinical use is in the surgical workup of medically refractory epilepsy to distinguish slowly progressing ambiguous and focal MRI lesions that may be caused by ganglioglioma or predominantly low-grade astrocytoma/oligodendroglioma from dysembryoplastic neuroepithelial tumour (DNT) or FCD. Distinguishing between different pathologies has a significant impact on patient care as it may set the indication for surgery, as the latter are at risk for malignant progression, while the former usually have a benign course. Furthermore, postsurgical seizure outcome is dependent on complete excision of the lesion, and AA PET may determine the glioma extension that usually requires a more limited removal than FCD, where a wider cortical resection using invasive electroencephalography may be recommended. At the group level, DNT and FCD show lower AA uptake than astrocytoma, oligodendroglioma, and ganglioglioma [113, 176, 177], but the literature is not sufficiently large to consistently separate the two groups [178, 179]. Approximately 30% of low grade glioma are inactive using [^18^F]FET [180, 181], and it may be in the same order for [^11^C]MET and [^18^F]FDOPA [182, 183], while some DNT and FCD show up to moderately increased activity [113, 178, 179, 184, 185]. However, there is agreement that a marked increased AA activity uptake strongly supports the neoplastic nature of a lesion. The report of a particularly favourable diagnostic accuracy of [^11^C]MET (AUC: 0.95) may be caused by a disproportionately large fraction of glioma with an oligodendroglial component [178], which are generally more active than the astrocytoma [186]. Single case reports using [^11^C]MET PET show marked uptake in chronic progressive encephalitis such as Rasmussen syndrome that may be associated with inflammation or epileptogenic activity and similar marked uptake in meningio-angiomatosis (Sturge-Weber syndrome) [187, 188].

### Serotonin receptor ligands

The 5-hydroxytryptamine receptor 1A (5-HT1A) receptor is a subtype of serotonin receptor located in presynaptic and postsynaptic regions. Several 5-HT1A receptor radioligands such as [^18^F]FCWAY100635, [^11^C]WAY100635, and [^18^F]MPPF have been developed over the years demonstrating a lower binding in the ipsilateral temporal lobe, which may help to identify the epileptogenic zone. Moreover, the overwhelming majority of studies also observed a reduced binding in the brainstem and limbic connections. The latter might explain the affective symptoms that can appear in patients with TLE. In contrast, an increased binding potential contralateral to the epileptogenic side in patients with TLE is described. This might be explained by differences in tracer affinity as [^18^F]MPPF is more sensitive to serotonin than [^18^F]FCWAY100635 and [^11^C]WAY100635 [189]. Additionally, ASM significantly influences the plasma free fraction of [^18^F]FCWAY100635 [190]. In summary, 5-HT1A receptor radioligand imaging is more of a scientific interest and has not yet been introduced into the clinical routine of epilepsy imaging.

### Other PET tracers

Numerous other radiotracers for different targets have been also used in epilepsy imaging research, such as synaptic density, neuroinflammation, neurotransmitter systems including the opioid system, but are so far not used in clinical practice.

#### Synaptic density

Synaptic vesicle glycoprotein 2A (SV2A) is a marker of synaptic density. Moreover, it is the binding site for levetiracetam, an ASM [191]. Lower SV2A has been detected in epileptic lesions both in TLE with medial temporal lobe sclerosis (37% ± 19%) using [^11^C]UCB-J and in FCD type 2 (27% ± 10%) using [^18^F]SynVesT-1 [192, 193]. The decrease in Sv2A is correlated with a reduction in [^18^F]FDG uptake.

#### Neuroinflammation

It has been demonstrated that epilepsy can cause neuroinflammation but also the other way around. Several studies reported an increase in translocator protein (TSPO) signal in the epileptic lesions, as proxy for neuroinflammation. Similar to synaptic density, the increased TSPO signal is associated with a with a reduction in [^18^F]FDG uptake [194–196]. Additionally, TSPO increases in the contralateral side have been described, illustrating both focal and distributed neuroinflammation [196–198].

#### Neurotransmitter

Besides GABA and serotonin, which are described above, other neurotransmitter systems have also been investigated in epilepsy. Glutamate transport has been examined using a radioligand for NMDA receptors, [^18^F]GE-179, and a radioligand for metabotropic glutamate receptor type 5 (mGluR5), [^11^C]ABP688. In focal epilepsy, clusters of increased [^18^F]GE-179 binding were found. [199, 200] In addition, global [^18^F]GE-179 binding was higher, except in patients taking antidepressant drugs in whom [^18^F]GE-179 was decreased [199]. In focal cortical dysplasia lower [^11^C]ABP688 binding was found in the lesion [201]. Dopamine neurotransmission has been investigated using multiple targets such as [^18^F]FDOPA (analog of levodopa), [^18^F]fallypride (D2/D3 receptors), [^11^C]SCH23390 (D1 receptor), [^11^C]raclopride (D2 receptor), and [^11^C]PE2I (dopamine transporter) with generally reduced uptake [202–208]. Increased [^18^F]FDOPA uptake was described in TLE in the epileptogenic region and increased [11C]raclopride uptake in the striatum and thalamus in myoclonic epilepsies [209, 210]; subcortical decrease of [^18^F]FDOPA uptake was found in various types of drug resistant epilepsy, including TLE [202, 203]. Lastly, nicotinic neurotransmission using the α4β2-nicotinic acetylcholine receptor ligand [^18^F]F-A-85380 has been investigated in autosomal dominant nocturnal frontal lobe epilepsy and in idiopathic generalized epilepsy, in which regional binding increases were observed [199, 211].

#### Opioids

There are three main opioid receptor types, µ, κ, and δ [212]. Post-ictal increases in the nonselective (µ, κ, and δ) radioligand [^11^C]diprenorphine in TLE were observed in ipsilateral temporal region relative to the interictal state [213, 214]. Similarly, the δ antagonist [^11^C]methylnaltrindole ([^11^C]MeNTI) and the µ agonist [^11^C]carfentanil had higher binding in the epileptogenic region in TLE and the κ and µ antagonist [^18^F]cyclofoxy was higher in complex partial seizures in mesial temporal lobes, thalamus, basal ganglia, and frontal cortex. [215] In reading epilepsy [^11^C]diprenorphine binding was decreased in reading-associated cortical regions during reading-induced seizures [216].

## Abbreviations

[^11^C]AMT: Alpha-[11C]methyl-L-tryptophan
[^11^C]FMZ: [11C]flumazenil
[^11^C]MET: L-([11C]methyl)methionine
[^18^F]FDG: [18F]fluorodeoxyglucose
AA: Amino acid
ASM: Anti*-*seizure medication
EANM: European Association of Nuclear Medicine
ECD: Ethyl cystine dimer
EEG: Electroencephalography
ETLE: Extra-temporal lobe epilepsy
FCD: Focal cortical dysplasia
GABA: Gamma-aminobutyric acid
HMPAO: Hexamethylpropyleneamine oxime
MCD: Malformation of cortical development
MRI: Magnetic resonance imaging
NM: Nuclear Medicine
PET: Positron emission tomography
SISCOM: Subtraction of ictal and inter-ictal SPECT co-registered to MRI
SOZ: Seizure onset zone
SPECT: Single-Photon Emission Computed Tomography
TLE: Temporal lobe epilepsy
